# Oral Sex, Oral Health and Orogenital Infections

**DOI:** 10.4103/0974-777X.59252

**Published:** 2010

**Authors:** Rajiv Saini, Santosh Saini, Sugandha Sharma

**Affiliations:** *Departments of Periodontology & Oral Implantology, Rural Dental College, Maharashtra, India*; 1 Department of Microbiology, Rural Medical College, Loni, Maharashtra, India; 2 Department of Prosthodontics, Rural Dental College, Loni, Maharashtra, India

**Keywords:** Oral Sex, Oral Health, Orogenital

## Abstract

Oral sex is commonly practiced by sexually active male-female and same-gender couples of various ages, including adolescents. The various type of oral sex practices are fellatio, cunnilingus and analingus. Oral sex is infrequently examined in research on adolescents; oral sex can transmit oral, respiratory, and genital pathogens. Oral health has a direct impact on the transmission of infection; a cut in your mouth, bleeding gums, lip sores or broken skin increases chances of infection. Although oral sex is considered a low risk activity, it is important to use protection and safer sex precautions. There are various methods of preventing infection during oral sex such as physical barriers, health and medical issues, ethical issues and oral hygiene and dental issues. The lesions or unhealthy periodontal status of oral cavity accelerates the phenomenon of transmission of infections into the circulation. Thus consequences of unhealthy or painful oral cavity are significant and oral health should be given paramount importance for the practice of oral sex.

## INTRODUCTION

Oral sex refers to sexual activities involving the stimulation of the genitalia by the use of the mouth, tongue, teeth or throat. Oral sex is now very common in both heterosexual and homosexual couples. People may involve in oral sex as part of foreplay before sexual intercourse, or during or following intercourse. Oral sex may be practiced by people of all sexual orientations. A significant proportion of adolescents are engaging in noncoital sexual activities, including oral sex.[1–5] Studies indicate that between 14% and 50% of adolescents have had oral sex before their first experience with sexual intercourse[3,5–8] that more adolescents have had oral sex than vaginal sex[5–9] and that few adolescents who engage in oral sex use barrier protection.[10]

The various types of oral sex performed are:

1. Cunnilingus (Oral Vaginal Contact): Oral stimulation of a woman's vagina and/or vulva, especially her clitoris, by her partner's lips and tongue
2. Fellatio (Oral Penile Contact): Stimulation of a man's penis by his partner's mouth-usually by licking or sucking.
3. Analingus (Oral Anal Contact): Stimulation of the partner's anus with tongue or lips

While the youth consider oral sex much “safer” than vaginal sex, this is a perilous fallacy. Although pregnancy is not an outcome of oral sex, sexually transmitted infections (STIs) are. Teens and the adults who involve in oral sex need to know that oral sex is associated with several STIs, including HIV. Oral sex is an efficient mode of transmission for syphilis,[11] gonorrhea[12,13] and herpes[14] HIV[15] Chlamydia[16] and HPV[13] can also be transmitted through oral sex.

## ORAL HEALTH PROFILE

The Surgeon General's report on oral health highlights the relationship between oral and overall health, emphasizing that oral health involves more than dentition.[17] Mouth acts as a window to lot of systemic diseases and serves as a port of entry of the various infections that can alter and affect the immune status of the person. The oral cavity has the potential to harbor at least 600 different bacterial species, and in any given patient, more than 150 species may be present, surfaces of tooth can have as many as billion bacteria in its attached bacterial plaque and oral care may not only reduce the microbial load of the mouth but the risk for pain and oral infections as well.[18] Good oral hygiene is the fundamental for oral integrity as it greatly affects the quality of life. Lesions of the oral cavity have an immense impact on the quality of life of patients with complex advanced diseases;[18] they cause considerable morbidity and diminish patients physical and psychological well being. The consequences of unhealthy or painful oral cavity are significant and oral health should be given paramount importance for the practice of oral sex. The good oral health permits in building up defense against the various viruses and organisms by obstructing their entry into body and circulation.

## ORAL HEALTH IN ORAL SEX

Although oral sex is infrequently examined in research on adolescents, oral sex can transmit oral, respiratory, and genital pathogens.[16] Oral-genital contact can transmit a number of sexually transmitted infections (STIs) including herpes, gonorrhea, and the human immunodeficiency virus (HIV).[19] In various acts of oral sex there is a risk of infection since saliva, pre-cum, semen, vaginal secretions, and menstrual blood can get into the mouth. The practice of oral sex is also highly prevalent among young people, regardless of whether they have previously engaged in penetrative intercourse[20] and more of these body fluids you are exposed to, the greater the risk of infection there would be. The various channels in oral cavity that serve as a gateway of entry of infection from oral cavity to blood stream includes any open sores, cuts, abrasions, or bleeding gum disease (gingivitis, periodontitis) in the mouth, the virus can get into the systemic circulation. The clinical depiction and silhouette of the various venereal diseases and infections spread through oral sex along with the possible channel of passage are mentioned in Table 1.

**Table 1:** Oral sex: Venereal diseases and infections

Infections/Diseases	Clinical picture and profile	Frequent transmission mode
Human immunodeficiency virus	Life threatening sexual transmitting disease. Hamper immune system especially CD_4_ cells. Secondary and super infection proceeds. No specific cure though HAART (Highly Active Antiretroviral Therapy) has some significant effects.	Cunnilingus, fellatio and analingus
Gonorrhea	Sexual transmitted disease. Sore throat. Burning sensation and discharge from penis. In extreme cases cause infertility and tubal pungency in women. Increases HIV load. Treatment with antibiotics under proper medical visualization.	Fellatio
Syphilis	Sexual transmitted disease. Easily passed through contact with open sores (commonly called chancres) on the penis, anus, or mouth (White spots in mouth) Sores, warts and rashes of syphilis infection are painless Left untreated syphilis can eventually cause brain damage, heart disease, blindness and death. Open syphilis sores or chancres provide an easy entry and exit for HIV and can increase viral load Antibiotic coverage and periodic medical check ups will be the line of treatment	Analingus, cunnilingus and fellatio
Chlamydia	STD caused by the *Chlamydia trachomatis* bacteria and affects women more than men. Common features include pain while urinating, smelly vaginal or penile discharge, spotting after intercourse, can be found in the throat but less commonly than gonorrhea. In extreme cases cause severe damage to women reproductive system, including permanent infertility Increases HIV viral load. Can be cured by proper medical treatment.	Fellatio, cunnilingus and analingus
Herpes	An STD caused by herpes simplex virus is the commonest cause of genital ulceration. There are two types of the virus; Type 1 affects mainly the lip causing cold sores and Type 2 causes blisters on the genitals. Sores and blisters (usually on the lips, genitals, or anus) are very infectious and painful. Research suggests that having genital herpes can more than double your risk for HIV infection. Some individuals with herpes usually have periodic outbreaks throughout their lives. Treatment can reduce the frequency and severity of herpes outbreaks but there is no cure.	Fellatio, cunnilingus and analingus
Human papilloma virus (Genital warts)	HPV infection and genital warts are the most common STDs. Warts usually appear on the penis or in the anus but may also occur in or around the mouth or lips. Genital warts may be more common and; harder to treat. Spread through skin-to-skin contact, contact with warts or HPV. While most strains of HPV only cause warts, some strains may cause oral or throat cancers. Different cures are available but the virus stays in the body.	All modes of oral sex
Non specific urethritis	NSU can cause burning when urinating and/or discharge from the penis. Infections of the throat can cause a sore throat. NSU may amplify viral load in semen making it easier to spread HIV. Manageable with antibiotics and hospitalization.	Fellatio, cunnilingus
Hepatitis A and E	Both these diseases can be spread through oral sex. Hepatitis A and E both are contagious viral infections of the liver. Common symptoms of hepatitis are fever, diarrhea Loss of appetite, dark urine, vomiting, jaundice and pain in the abdomen. Vaccination is available for prevention	Analingus
Hepatitis B	It is most commonly transmitted by inoculation of infected blood, virus particles are found in semen, stool and saliva, as well as blood. There is clear evidence that it can be transmitted through vaginal and anal intercourse, but it is unproven whether it can be transmitted through oral sex. Hepatitis B can cause weakness, dark urine, jaundice (yellowing of skin and eyes), and enlarged liver. Vaccination is available for prevention	Fellatio, cunnilingus and analingus
Bowel organisms and worms	The bowel organisms *Salmonella*, *Shigella* and *Campylobacter* can all be transmitted. Abdominal pain and diarrhea Treated well after microbiological stool examination	Analingus
Intestinal parasites	These include *Amoeba*, *Giardia* and *Cryptosporidia*. Symptoms include Unknown diarrhea, stomach cramps, bloating, increased gas, and nausea. Treated well after microbiological stool examination	Analingus

## SALIVA, TEETH AND HIV

The potential for transmission of HIV by saliva is low, probably due to the low levels of infectious virus and potential HIV inactivating agent(s) in saliva.[21] The unique combination of a thick epithelial layer, reduced numbers of CD4-bearing target cells, antiviral antibodies and several endogenous inhibitors (including SLPI) make the oral cavity a particularly resistant site for HIV transmission. Nonetheless, antiviral mechanisms are not impermeable, particularly if HIV is delivered as a bolus (as in receptive oral sex) or the integrity of the mucosal surface is breached (as with tears, lesions or periodontal disease.[22] The intact mucosal membrane constitutes a formidable barrier to infection by pathogenic microorganisms, including viruses. In addition to lubricating mucosal surfaces; saliva dilutes the microbial burden and flushes microorganisms into the gastrointestinal tract for inactivation and destruction.[22] Dentinal carious lesions may serve as a reservoir for Candida organisms in both HIV-positive and HIV-negative people, but they are more common in HIV infected people and may participate in recurrent or recalcitrant oral Candidiasis in immunosuppressed or immunocompromised patients. The eradication of dentinal carious lesions with tooth restoration or extraction, when indicated, may eliminate potential fungal reservoirs responsible for recurrent or recalcitrant clinical oral candidiasis.[23] Current evidence suggests that the risk of HIV transmission from exposure to saliva is considerably smaller than the risk from exposure to semen.[24]

## INFECTION PROGRESSION AND PREGNANCY

The biologic risk for transmission or acquisition of HIV from oral sexual contact is not known, but the risk is likely to be related to a number of factors. These include the presence or absence of virus at sexual sites (oral, vaginal, anal and penile), the titer of virus (if present), the integrity and mechanical properties of the sexual mucosa, mucosal immunity, local inhibitory factors, and the presence or absence of cofactors that may facilitate transmission. Finally, the frequency and nature of exposure (e.g., the relative effect of a large number of lower risk events compared with a small number of higher risk events) and the underlying epidemiologic features of HIV dynamics in the community may have an impact on the frequency of HIV transmission from oral intercourse.[24] The presence of chronic conditions, the occurrence of chronic ulcerating lesions (candidiasis, herpes simplex virus infection, apthous ulceration, ulcers secondary to crack cocaine use), and the presence of many oral pathogens may provide an opportunity for facilitation of HIV transmission similar to that which occurs with sexually transmitted diseases. Similarly, the proportional importance of oral sex to HIV transmission will be a complex result of the relative frequency of oral sex compared with other activities, infectivity of oral secretions and its modification by oral pathology, resistance to infection by inhibitory substances in saliva, the HIV prevalence in the community in which such activity takes place, the maturity of the epidemic in the community (given recent observations on differential infectivity by stage of infection, the role of high activity antiretroviral therapy, and the extent to which personal prophylaxis is adopted).[24]

Oral sex with ejaculation was perceived as more risky than oral sex without ejaculation, across scenarios, receptive anal intercourse was judged to be riskier than insertive anal intercourse, which was perceived as riskier than oral sex.[25] The evidence suggests that HIV transmission can take place through oro-genital sex from penis to mouth and vagina to mouth. Case reports describe apparent transmission from mouth to penis although this appears less likely. The risk of oro-genital transmission of HIV is substantially less than from vaginal and anal intercourse. Receptive oro-genital sex carries a small risk of human papillomavirus infection and possibly hepatitis C, while insertive oro-genital contact is an important risk factor for acquisition of HSV 1. Oro-anal transmission can occur with hepatitis A and B. The transmission of other viruses may occur but ha not yet been proved. The relative importance of oral sex as a route for the transmission of viruses is likely to increase as other, higher risk sexual practices are avoided for fear of acquiring HIV infection.[26] Thus, unprotected oral-genital contact was the most commonly reported sexual activity in patients who developed primary HIV infection. Increasing attention to the risks of oral-genital contact as an important means of HIV acquisition appears to be warranted;[27] although it is true that oral sex negates the risk of pregnancy[10] STI is an issue. There is no pathway or scope for sperm from the penis to enter the uterus and fallopian tubes to fertilize an egg. In humans, there is no connection between the gastrointestinal system and the reproductive tract. Ingested sperm is killed and broken down by acid in the stomach and proteins in the small intestine. The breakdown products will be absorbed as a negligible quantity of nutrients. Despite this, oral sex does carry a possible risk of pregnancy if semen from the man comes in contact with the vaginal area circuitously. This can occur if the semen in the ejaculate is carried on the fingers, hands, or other body parts; and comes in contact with the vaginal area. It is therefore still essential to exercise awareness when having oral sex to avoid pregnancy.

## SEXUAL PRACTICES AND BEHAVIOR

The risk of obtaining an STI through oral sex is certainly lower than the risk of infection through sexual intercourse; research has indicated that oral transmission is an important health concern, particularly because some adolescents and adults erroneously view oral sex as a risk-free behavior. However, substantial changes in attitudes and social norms may be required before there are noticeable differences in teen use of protection for this relatively low-risk sexual behavior; indeed, many teens may purposefully engage in oral sex to avoid the greater risks associated with other sexual behaviors.[28] The findings suggest that in studying oral sexual behavior we need to be aware of the interaction of social, relationship, and attitudinal variables as well as the relationship of oral sexuality to other sexual behaviors. The increased reporting of risky sexual behaviors is consistent with changing cohabitation patterns and rising incidence of sexually transmitted infections.[29] An individual's choices of partner and sexual behavior are based on both the risk of acquiring an infection and the benefits derived from the sexual relationship. When multiple acts over a period of time are considered, frequency of sex and number of partners are important contributors to cumulative risk. In this context, choosing safer sex acts could lead to other behavior changes that increase risk. For example, oral genital contact may be less efficient at HIV transmission than other sex acts, but if oral sex is practiced more frequently or with risky partners (because it is perceived to be safe), it could increase the risk for HIV infection, similarly, having a larger number of partners increases the likelihood of exposure to an infected or highly infectious partner.[30]

Actively manipulating social norms and adolescents' perceptions of the social benefits associated with sexual behavior may also prove to be effective prevention strategies.[28] Evidence for the occurrence of HIV transmission through oral sex is becoming clearer with the shift away from higher risk sexual behavior. The main dilemma now is how to present the small but real risk of oral sex without encouraging a resumption of higher risk sexual activity (including anal intercourse), which it has been suggested may accompany an awareness that oral intercourse is not risk-free.[31]

## SAFER ORAL SEX

Due to above mentioned disease risk, it is advisable to use proper precautions when performing or receiving oral sex with a partner. It is not as risky as unprotected anal or vaginal sex, but it is still possible to get HIV and other venereal diseases and infections in this way. There have been a few documented cases of HIV transmission this way. HIV is found in blood, semen (cum), vaginal fluids, and breast milk. The virus can transmit through cuts, openings, sores, and mucous membranes (mouth, anus, and vagina) to the body. The various manners to minimize the chances of getting infection during the oral sex are illustrated in Table 2.

**Table 2:** Methods of preventing infection during oral sex

Physical barrier and precautions
Latex/polyurethane condoms (flavored condoms are available) during fellatio Dental dams/cut-open condom/latex squares during cunnilingus Plastic wrap (If approved) during analingus
Health and related issues
Avoid oral sex if any of the partners has Any sexual transmitting diseases/infections (STD/STI) Wounds or open sores on their genitals Wounds or open sores on or in their mouth, or bleeding gums Avoid oral sex with women during menstrual periods Genitals, as well as the surrounding area, is washed and cleaned thoroughly.
Ethical and social issues
Limiting the number of your sexual partners Avoiding ‘casual’ sex with an unfamiliar partner Do not feel pressure to have oral sex Practice safe sex by using a condom every time you perform oral sex
Oral hygiene and dental issues
Wait to have oral sex at least 30 minutes after brushing or flossing your teeth Avoid oral sex after recent dental treatment or periodontal therapy (Dental scaling and periodontal surgery) Elude of getting body fluids in mouth/keeping in mouth for longer duration, if occurs rinse mouth with antibacterial mouth washes
Medical screening and education
Regular health checkup and screening especially dental check up If any doubt or uncertainty it is essential to seek medical advice as soon as possible and talk to professional for more information Effective treatment is available for most diseases, including HIV Early treatment is very important Sex education

## PREVENTION AND BARRIER TECHNIQUES

To avoid risks during oral sex it is advisable to keep semen and vaginal fluids out of mouth as earliest. The oral cavity should free from any potential bleeding tendencies or pathology. Due to disease risks, many medical professionals advise the use of condoms or dental dams when performing or receiving oral sex with a partner whose STD status is unknown. A makeshift dental dam can be made out of a condom. Using a real dental dam is preferable, because real dental dams are larger and the makeshift version may be accidentally poked with the scissors during the cutting procedure. Plastic wrap may also be used as a barrier during oral sex, but many find that the thickness of the plastic dulls sensation. Details of various methods and technique are illustrated in Table 3.

**Table 3:** Preventive and barrier techniques

Involvement	Barrier methods	Characteristics	Drawbacks	Directions
Oral sex on vulva and anus	Plastic wraps	Inexpensive and easy to locate Covers large area Lubricated if required More pressure sensitive	Chances of torn by finger nails Slip up during the sexual course Aggressive sexual act may torn the plastic wrap	Cover the vulva area with the plastic wrap. Either cut a piece of the wrap and hold it in place or wrap the pelvic area Add lubricants for more sensitivity and sexual pleasures After the act discard the wrap safely
	Dental dams/latex square barriers	Provides a strong latex barrier Lubricated and flavored can be used	Covers a small area and fluids may seep past the dam May not be used with oilbased lubricants because they will break down the latex Less sensation of warmth and feeling Not easily available	Hold the latex square over the vulva area Sensitivity can be increased by lubricant on the side facing the vulva Single use for one act
	Cut condoms	Non lubricated condom, flavor lubricated condom or flavored non lubricated condom	Provides a small area of protection and care to ensure that fluids don't seep past the condom into the mouth or the anus/vulva area Use water-based lubricant Prevents effectively if placed properly	Unroll the condom and cut off the very tip and the very end of the condom and cut lengthwise to make a rectangle Hold the latex square over the vulva area Water-based lubricant (not Vaseline or oils) can be used for increasing sensitivity During rimming place the condom over the anus Single time use per sexual act
Oral sex on penis	Condoms	Non lubricated condom, flavor lubricated condom or flavored non lubricated condom Safe and best method Easily available	Protects what it covers Avoid slipping of condom Placed properly over the lips or between the lips to prevent sharp cut by teeth	Condom to be uploaded properly Single use Avoid aggressive sucking to prevent slippage Different flavors as per partners choice

## CONCLUSIONS

The practice of oral sex is also highly prevalent among young people, regardless of whether they have previously engaged in penetrative intercourse.[20] Oral sex involves giving or receiving oral stimulation (i.e. sucking or licking) to the penis, the vagina, and/or the anus. However, although the risk of STD transmission is far greater during vaginal and anal sex than during oral sex, the increasing practice of oral sex, low rates of barrier method use and the finding that first oral sex often occurs prior to first vaginal or anal sex will help increase the relative importance of oral sex as a mode of transmission for genital pathogens.[13,16,20] HIV, other STDs can be transmitted through oral sex with an infected partner examples of these STDs include HIV, herpes, syphilis, gonorrhea, genital warts (HPV), intestinal parasites and hepatitis. There are several ways to reduce the risks of oral sex. Generally, the use of a physical barrier during oral sex can reduce the risk of transmission of HIV and other STDs. To reduce the risk of infection during unprotected oral sex, limit exposure to sexual fluids and ensure that no cuts or lesions are present in mouth or on genitals. A good oral health, free from bleeding gums, lip sores, cuts, broken skin and oral epithelium enormously reduces the chances of transmission of infection among the partners indulge in oral sex. A periodic oral health check up is mandatory among the people frequently involved in oral sex and thus good oral hygiene is the fundamental for oral integrity as it greatly affects the quality of life.[18]
